# Why the history of public consultation matters for contemporary health policy

**DOI:** 10.1016/j.endeavour.2018.01.001

**Published:** 2018-03

**Authors:** Jennifer Crane

**Affiliations:** Centre for the History of Medicine, University of Warwick, United Kingdom

**Keywords:** NHS, Consultation, Health policy, Public opinion

## Abstract

Contemporary policy debates construct public involvement in England’s National Health Service as “new,” or as a practice dating back only as far as the 1990s. This article argues that the longer historical contexts of such consultative practice matter, and it explores various and shifting manifestations of “consultation” in the NHS from the foundation of the Service in 1948. In doing so, it first demonstrates that consultation has always been a part of the theory and practice of postwar health policy. Thinking about consultation as “new” presents such practice as unnecessary or transient, and may function as part of a damaging political vision of public affection for the NHS as a barrier to reform. Second, the article asserts that public interest in shaping NHS practice and policy has never been fully satisfied by official consultative mechanisms. “The public” is not a homogeneous group, but rather composed of various groups, communities, and individuals with rich perspectives and histories to share, having experienced the NHS as patients, friends, supporters, staff, and volunteers. Policy-makers should approach diverse publics as partners, and should meaningfully listen to protests around NHS reform, which often reflect public investment in the NHS, as well as valid concerns about how particular communities will be able to access health care. While the political will for such engagement has varied over time, individual politicians and local-level health agencies can make a difference by supporting, engaging with, and funding organizations which represent and empower a diverse range of communities: such groups have always, and will continue to play, a significant role in shaping NHS debate and care.

## Highlights

• Public consultation in England’s National Health Service is not new, but dates back to the foundation of the Service, in various forms.
• Public will to contribute to NHS policy has never been constrained within “official” consultative practice alone.
• Policy-makers should empower and listen to public perspectives on NHS policy.

“Whose NHS?,” “Our NHS!”

*Chant during 250,000-strong march in defense of “Our NHS,” London, March 4, 2017*

“One of the great strengths of this country is that we have an NHS that—at its best—is ‘of the people, by the people and for the people’ … we need to engage with communities and citizens in new ways, involving them directly in decisions about the future of health and care services.”

*NHS England, The Five Year Forward View, 2014*

**Figure 1:**
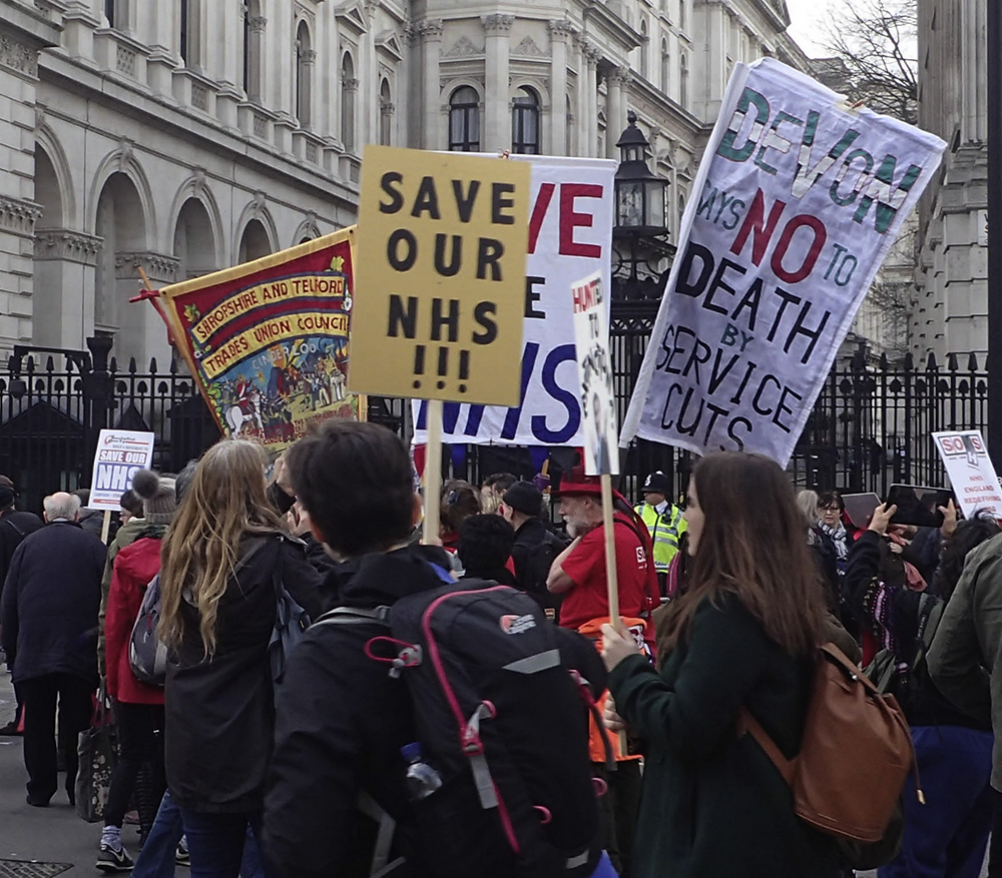
By Gwydion M. Williams from Coventry, Great Britain (2017_03_040044d) [CC BY 2.0 (http://creativecommons.org/licenses/by/2.0)], via Wikimedia Commons.

**Figure 2:**
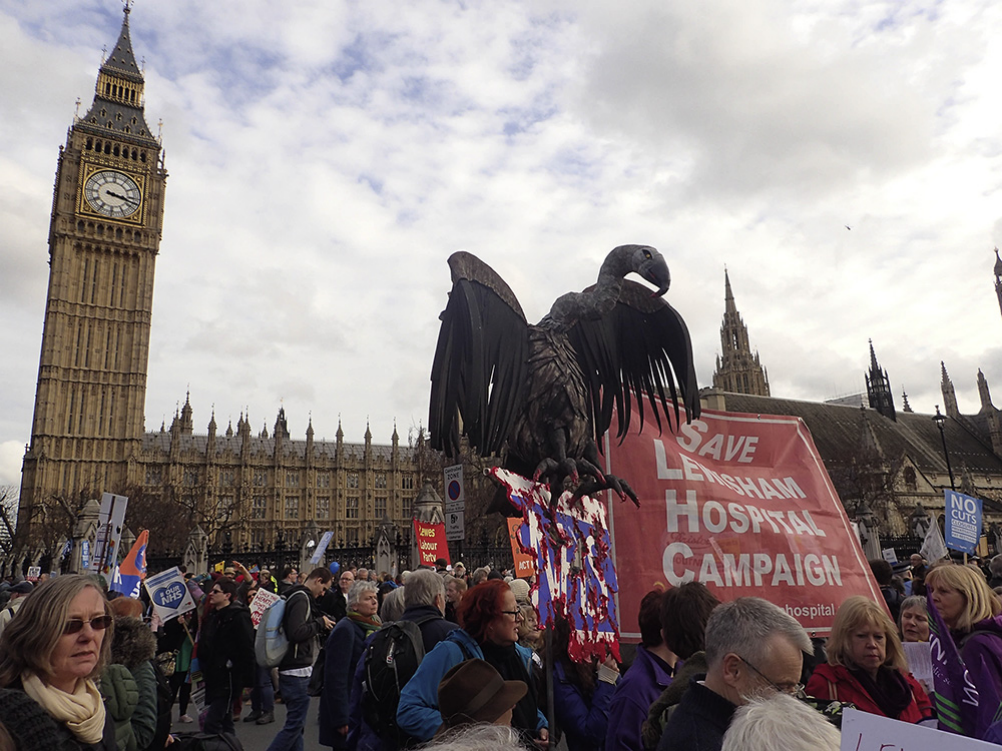
By Gwydion M. Williams from Coventry, Great Britain (2017_03_040052) [CC BY 2.0 (http://creativecommons.org/licenses/by/2.0)], via Wikimedia Commons.

Tens of thousands of people flooded into Parliament Square in London in March 2017, enraged by cuts, closures, and private provision in the UK’s National Health Service. Convinced that their voices had not been heard in discussions over reforms to “our NHS,” (Figure 1, Figure 2) the protesters displayed their level of commitment to the service. They had reasonable grounds to do so. The Five Year Forward View, a 2014 strategy document published by NHS England, stated that the National Health Service belongs to “the people,” and articulated the importance of engaging with “communities and citizens” during health policy planning. The march nevertheless indicates that broad swathes of the public feel that their interests are not being represented during health reform, notably following a large-scale reorganization of the service in the 2012 Health and Social Care Act and as, the following year, NHS England stated that even if government spending on the service continued in line with inflation, it would still face a funding gap of £30 billion by 2020–2021.^1^

Despite public “involvement,” “consultation,” or “listening” exercises, then, a significant proportion of the population still feels the need to defend the service, most recently against major changes to NHS financing and provision advocated by Conservative Prime Minister Theresa May and her predecessor, David Cameron. Concerns about NHS funding are no minority position: in a recent sample, two-thirds of the population told pollsters that they would like to contribute further tax for the NHS.^2^ The evident failure of policy to meet these public expectations raises questions about what policymakers have wanted and expected from consultation exercises, and the limits of consultative practices—which aspects of NHS policy and practice have, and which have not, been up for public debate, and why? When is public opinion influential, and when is it ignored? In this context, to what extent do members of the public trust consultation exercises, and how does this reflect broader senses of trust or distrust in government oversight?

Looking to history can help us think through these questions, seeing how they have been formed and how different Governments and public groups have responded. The complex interface between publics and policy in this area has developed since the NHS was founded in 1948 by a postwar Labour government. Despite growing public and political interest in “consultation” through the late twentieth century, the term has meant very different things to different groups. Political consultative exercises have sometimes been tokenistic, and have not always captured the depth, significance, and richness of public feelings about the NHS. Consultative exercises have at times been hindered by political visions of publics as a barrier to NHS reform, rather than as a partner in promoting health.

Looking historically suggests that an array of voluntary organizations have played a key role in driving and enabling public involvement in NHS planning and practice, often by operating outside of “official” consultative mechanisms. At the inception of the NHS itself, medical interests, organized through trade and labor unions, were significant. From the 1970s, new mediatory bodies emerged to unite, and speak on behalf of, particular public constituencies: Community Health Councils organized geographical communities; patient advocacy groups represented individuals concerned about particular medical conditions; and new voluntary political groups represented those opposed to, or supportive of, local and national reforms. Effective organizations have represented vulnerable populations, and enabled a broad range of individuals to share their experiences of NHS care publicly, despite financial, temporal, and emotional barriers to individual-level political action. NHS policy would best represent publics by consulting with and empowering a broad variety of such mediatory groups, and by supporting new such groups to flourish to represent a diverse range of communities. Although the political will for this endeavor is not always present, such groups have nonetheless had significant successes in shaping and contesting local and national reforms since the inception of the NHS.

## “Those concerned … shall be fully consulted”: What Was Public Consultation?

### Consulting Consultants? The Early NHS

Building on early twentieth century research from liberal and social progressives, and on local experiments and insurance schemes, political discussions about creating a nationalized health service in the UK developed in earnest during World War II.^3^ The report *Social Insurance and Allied Services* (1942) proposed the implementation of a new, universal health care system to sit alongside new systems of family allowances, national insurance, pensions, and unemployment benefits. The report was commissioned by government and written by a temporary wartime civil servant, William Beveridge. Contrary to expectations for such a bureaucratic document, over 600,000 copies were sold by February 1944. What became known as the “Beveridge Report” was widely discussed on radio, in press and, social surveyors found, across British society.^4^ The popular appeal of this report demonstrated the strong public appetite for such a service to the political parties.

Even though public interest was visible in this way, accessing and mediating the interests of medical professionals, rather than those of the public, was the priority of early debates around the NHS. In 1944 the Ministry of Health, led by Conservative Henry Willink, published the white paper, *A National Health Service*. The paper promised that “those concerned, professionally and otherwise, shall be fully consulted before final decisions are taken.” The document emphasized that the Government would welcome “constructive criticism” and “public discussion,” but also expressed hopes that these would “enable them to submit quickly to Parliament legislative proposals which will be largely agreed.” The document’s focus on getting feedback from “professionals” rather than those categorized as “otherwise” was clear. It emphasized that the Government had already had discussions with local authorities, the medical profession, and voluntary hospitals, which were “considering the form which the new National Health Service should take.”^5^

The new Labour government, elected in July 1945, set Willink’s specific plans aside. Labour’s Minister of Health, Aneurin Bevan, believed that central government should play a much more significant role in the new service than had previously been conceptualized, and also that all hospitals must be brought under public ownership. Bevan circulated new plans in January 1946 to organizations including the British Hospitals Association and the British Medical Association. Geoffrey Rivett argues that these plans were shared, “not so much for the purpose of consultation, but to prepare people for what was to come,” but Bevan nonetheless encountered opposition from these groups.^6^ The British Hospitals Association questioned whether these plans would weaken connections between local publics and their hospitals.^7^ The British Medical Association initially proved strong critics, arguing that doctors should hold independent status, rather than being state employees on fixed salaries.^8^

Following the publication of the National Health Service Bill in March 1946, however, other professional bodies expressed support for the new service. This was the position of the Socialist Medical Association (SMA), a campaign group affiliated with the Labour Party and open to membership to all health professionals. The SMA had, since its inception in 1930, called for a socialized medical service, and in 1946 argued that the NHS would be an “infinitely better health service than the present worn-out patchwork.”^9^ The Medical Practitioners Union, likewise, and having challenged Willink’s 1944 model of the NHS, circulated a letter to its members in January 1948 arguing that with the “co-operation of the profession in operating and perfecting it, the National Health Service Act can bring inestimable benefit to the profession and to the community.”^10^ Thus, although successive Ministries of Health expressed levels of interest in soliciting input about the new NHS, professional groups and organizations were the key focus of external consultation. External consultation continued while Ministers also sought consensus from their political colleagues in Cabinet and opposition, as well as in local government.

This is not to say that public voices were absent from the formation of the NHS. Certain members of the public, particularly from the middle and upper-classes, directly sat on Regional Boards and hospital management committees. A public guide to the new NHS emphasized that these people were more broadly, “non-professional men and women who are interested in the hospitals and in the good of their own communities,” and also highlighted the key role of volunteers in contributing to hospital life.^11^ Assumptions about public belief also influenced political thinking: indeed, the Medical Practitioners Union told its membership in 1948 that the introduction of an NHS was inevitable, not only because “the profession recognises its need” but also given that “the people welcome it” and “no political party dare support its postponement.”^12^

Again suggesting the import of public opinion during early discussions about the NHS, the BMA also sought to ally popular thinking to their cause. The organization’s secretary, Charles Hill, wrote to the *Daily Mail* in August 1945 to couch his arguments in terms of public accountability, stating, “Doctors want to be employed by their patients, to be responsible to their patients and to them only.”^13^ In March 1946, he wrote again outlining his critique of Bevan’s plan, and urging readers: “See to it that you have your say. See to it that your voice is heard.”^14^ Significantly, then, from the inception of the NHS, public voices were heard in a variety of ways, and medical representatives sought to understand, respond to, and shape public feelings. At times, however, policymakers and practitioners tended to imagine public preferences, rather than gleaning them directly.

Of course, not all of the public wanted to be actively involved in shaping new services. Social investigators at the inception of the NHS found people in East London taking a “perverse pride” in their disinterest in welfare reform, while the majority of those interviewed in Manchester “just did not know.”^15^ Media coverage reflected this lack of public concern, providing little newspaper coverage in the first days of the NHS’s operation.^16^ Following a brutal war, many members of the public were focused on reconstructing their lives. Others still were happy to accept the new service without calling for change, given that it replaced a system in which women and children in particular did not have easy access to health care. At the same time, the lack of direct public involvement in this period also reflected the ways in which consultation was dominated by the most organized interests, and in which public groups were not being specifically established in order to lobby for and shape the new service. At times, organized medical interests reflected and overlapped with broader public views; however, without direct consultative fora or mediatory bodies, few members of the public themselves could directly contribute to debate.

### Consulting Consumers

An expectation that NHS reform should involve widespread and direct public consultation emerged in the 1960s and 1970s. Created while local government and social services were also being reformed, the *NHS Reorganization Act* of 1973 marked the first significant restructuring of the NHS.^17^ The act reformed the tripartite structure created in 1948, whereby primary care, secondary care, and local public health services were managed separately. Hoping to improve coordination and efficiency across health services, the act created a new administrative system whereby health services were administered through geographically based authorities (divided by “region” and then, further still, by “area”).

Public concern about this reform was relatively minimal, and the BBC were reluctant to discuss the act on radio or television, suggesting that, “the public will not be interested.”^18^ However, the act itself created a new mechanism through which invested publics could seek to shape health care provision in the future: establishing local-level Community Health Councils (CHCs), covering populations from 86,000 to 530,000 people.^19^ CHCs were to be funded by the Regional Health Authorities, who paid for full-time staff (most had two), office space, and expenses.^20^ CHCs had around twenty to thirty board members, who were appointed for four years and could serve a maximum of two terms. Of this membership, half were appointed by local authorities—primarily councilors from the political parties—one-third were chosen by eligible voluntary organizations, and the remaining one-sixth were selected by the Regional Health Authority.^21^

Although the act stated that CHCs would “represent the interests in the health service of the public in its district,” it did not define precisely how they would undertake such work in practice.^22^ Accordingly, how CHCs operated varied across areas, shaped by perceptions of local needs and the character and preferences of their board members. Many focused on understanding local views about health services and identifying gaps. Another more confrontational form of action taken by CHCs sprung from their legal right to be consulted about hospital closures, and involved escalating opposition to closures to the courts.^23^ In 1979, for example, health service commissioners suspended in-patient services in St Olave’s Hospital, in South London. The local CHC argued in the High Court that there had been inadequate consultation, whereas commissioners testified that expenditure needed urgent reduction, so the suspension was necessary.^24^

The closure of this hospital was contested in the House of Commons, as well as in the courts, where the local Member of Parliament credited the “magnificent work” of the CHC, and underscored the strength of local feeling towards the hospital, “in the heart of my constituency,” as well as emphasizing its long history as an old infirmary.^25^ St Olave’s Hospital did not reopen, and indeed legal rulings in the mid-1980s suggested that health authorities ultimately had the power to close hospitals without consultation in cases of financial urgency.^26^ Nonetheless, CHCs continued to contest hospital closures in the courts, challenging the power of local authorities by using definitions of “consultation” constructed in state legislation.

CHCs were significant in opening up health policy to public voices in two key ways. First, the organizations reflected and contributed to a growing assumption within health policy work: that vulnerable public voices must be considered in reforms. Alex Mold has argued that CHCs tended to see themselves as guardians of community interests, rather than direct or representative spokespeople.^27^ Of the voluntary sector board members, one in ten worked for organizations concerned with mental health and a quarter worked on behalf of elderly people.^28^ People concerned with the interests of vulnerable groups were thus formally brought in to conversation with long-standing actors in health reform. The second key way in which CHCs were significant was in terms of more broadly disrupting assumptions about how health policy should be formed, and who should be involved. Many CHCs sought to challenge their vision of the NHS as a “closed system … largely run for the benefit of the doctors.”^29^ Through their existence and their actions, CHCs opened up a space in which members of the public could—and indeed felt like they should—seek to influence health policy.

This space was filled by a broad variety of public action, magnified during the Governments of Prime Minister Margaret Thatcher from 1979, which sought to reduce expenditure on the NHS, weaken trade unions, and enable private contractors to take over NHS services including cleaning and catering. In 1990, Thatcher’s government introduced the internal market, whereby health authorities and providers would purchase health care from hospitals and health organisations, a “purchaser-provider split.” Amidst these highly controversial changes, new voluntary groups emerged from trade unions, pensioner groups, local political parties, tenants’ associations, and patient organizations, all seeking to thwart particular local closures as well as national-level change. These groups often expressed their views through strikes, occupations, marches, lobbies, and petitions, rather than through state-funded consultative exercises or CHCs.

Groups such as London Health Emergency, formed in 1983 and initially funded by the Greater London Council, also focused on compiling detailed reports about the state of the NHS, drawing on official figures from Government departments and providing information and analysis to media and politicians.^30^ New patient groups provided another type of subversive challenge: confronting biomedical models of health by emphasizing the significance of social and psychological support, provided by peers as well as clinicians. These groups also rejected narrow avenues for reform, instead expressing their views through peer meetings, intimate media interviews and literary accounts. In addition to such collective action, individual families launched legal challenges against hospital closures in the 1990s, which continued to rely on the argument that local authorities had not followed statutory consultation procedures.^31^

Tensions around the meaning and limits of “consultation” continued throughout the late twentieth and early twenty-first centuries. The *Health and Social Care Act* of 2001, for example, reiterated a general duty of NHS organizations to involve and consult people in planning or changing health services. Yet this legislation did not define the level at which the public must be involved, which left space for further legal confrontation. Famously, in 2006, a pensioner named Pam Smith successfully challenged the right of United Health Europe, a subsidiary of the American healthcare provider United Health, to provide general practice services in her local area. Smith’s concern was that private providers would seek to profit out from NHS services. Her legal claim, however, was based on highlighting the low quality of public consultation conducted before this decision was made.^32^

Thus, although public consultation became increasingly relevant to NHS reform legislation beginning in the 1960s, public responses to reform were not narrowly contained within state-organized consultative exercises. Particularly during fundamental changes to the structure of the NHS in the reforms of 1974 and under Margaret Thatcher, members of the public used a variety of means to make their opinions heard. Many members of the public deployed state-produced knowledge to challenge NHS reform, for example by providing new analyses based on Department of Health statistics, or by using the consultative legislation to challenge health authorities in the courts. The state-funded CHCs, also, often provided a disruptive challenge to existing processes of negotiation between national governments, local health authorities, and organized medical interests, inspiring new actors to contribute to health policy debate.

### Progress?

It is tempting to trace a shift from 1945 through the early twenty-first century, during which NHS-related organizations were increasingly mandated to, and even became more interested in, consulting with the public. The relationships between publics and the health service have indeed shifted immensely over this period, most significantly perhaps in terms of relations between clinicians and patients: the early 1960s model, wherein clinicians would often tell patients little about their conditions, has declined.^33^ However, in terms of public involvement in the construction of policy, no linear, progressive narrative dominates. Policymakers continually remade their visions of patients in the NHS, first in the 1960s and 1970s in terms of rights and representation and later, under 1980s and 1990s Conservative governments, around markets and choice.^34^

Public consultation in the NHS shifted not only in line with these broad visions of “the public,” but also in pragmatic response to political contexts and perceptions of previous consultative challenges. Thatcher’s governments introduced the internal market to the NHS with minimal consultation and despite bitter opposition from public and medical professionals, who believed that making hospitals self-governing would lead to privatization, and who questioned how the GP-patient relationship would be affected by GPs becoming fund-holders.^35^ Health Minister Kenneth Clarke’s determined implementation of this reform may be contrasted to the laborious consultative exercises conducted by a previous Conservative government while designing the *Reorganization Act* of 1973, who sought “to achieve a consensus by consulting everyone and seeking to satisfy all interests involved.”^36^ The contrast between how these reforms were implemented demonstrates that the will from political administrations for consultation has retreated, as well as developed, over time. Political interest in consultation shifts depending on a Government’s power in the Commons (the 1989 Conservative government was working from a much stronger majority than the 1973 one); the specific style and beliefs of each Health Minister; and the current performance of the NHS, with crises acting as a political justification for urgent reform. Individual and institutional memories of previous consultative efforts are also important: in the 1989 case, many politicians believed that the consultative work of the 1973 reform had created an overly complex and unworkable system.^37^

Although the practice, efficacy, and meaningfulness of consultation have not necessarily increased over time, the usage of the term has. It is a broad term, with varying definitions imposed by patients, publics, and policymakers over time and space. Looking back to the inception of the NHS can further our understanding. Taking a longer historical view suggests that various public groups have felt increasingly empowered to assert their “rights” to influence health care reform, in a variety of ways. At the same time, demanding change has required that these groups commit serious of financial, temporal, and emotional resources to their cases, often at times when they themselves have faced significant health-related challenges.

Voluntary organizations have acted as mediators, amplifying individual voices. The press, the Socialist Medical Association, and various trade unions and campaign groups have, since the start of the NHS and before, shaped the ways in which the public have called for NHS reform, enabling certain people to influence reform whether through filling out surveys or by becoming spokespeople themselves. Community Health Councils and their later equivalents—Public and Patient Involvement Forums and local involvement networks—were state-funded efforts to manage this consultative process, but these fora also at times sharply challenged national governments and local health authorities. While intending to provide new spaces for patient voices, these groups also again required publics to develop a level of understandings of their internal systems and regulatory roles. Thus, history shows that everyday spaces of encounter have shaped the multiple relationships between policy and publics. These broad groups have engaged with one another not only during consultative exercises, but at the ballot box, through the informing and reading of opinion polls, and within the mediatory work of small but significant voluntary groups.

## Improving Involvement

The interface between publics and policymakers has thus not developed in a progressive manner, but rather has been recreated and reinscribed over time on local, regional, and national levels, and through various points of legal, medical, and political encounter. Public attempts to inform NHS debate have at times progressed separately from political efforts to track public opinion, with great frustration on both sides. One key barrier to change has been suspicion from politicians about the utility of consultative efforts, driven by a constant fear that public affection for the NHS will act as a barrier to improvement.^38^ This notion is visible, for example, in a newsletter of the Conservative Medical Society from the 1988, suggesting that the NHS’s “popularity” must not “obscure the need to find solutions to its genuine problems.”^39^ Tony Blair, reporting to the House of Commons Liaison Committee in 2006, similarly positioned public feeling as a barrier to change. Blair stated that even if clinicians advised that hospital provision should be centralized, local people would still protest. Suggesting that other politicians shared his attitude, Blair emphasized that “everybody who has ever dealt with a potential hospital closure” knew this “problem.”^40^

Policymakers have been particularly dismissive of campaigning, rallies, and marches—the popular spaces through which members of the public have sought to challenge or influence reform. In one retrospective witness seminar, senior civil servants and public inquiry members described campaigning as “downright insulting” and “table banging,” positioning such efforts as in conflict with “the greater good.”^41^ But not all political figures have shared this attitude. Prominent Labour Party Members of Parliament, including the party’s current leader, Jeremy Corbyn, attended and spoke at rallies against NHS cuts and closures in the 1980s.^42^ Throughout the late twentieth century, individual campaigners, campaign groups, and patient representatives established productive working relationships with individual Members of Parliament from all political parties.

Despite these positive encounters, however, much evidence remains that campaigners and publics have felt frustrated by NHS consultative practice. In 1983, one campaigner wrote to the Politics of Health Group that activists regularly encountered the same “slippery-tongued, public-school NHS yes-man.”^43^ Those creating petitions, such as Janet, who collected 180,000 signatures in 2002 against a local hospital closure, nonetheless often feel that “in the corridors of power, barely an eyelid was batted.”^44^ Despite the disillusion in these testimonies, however, and the suspicion of media and political interests, the individuals involved were still willing to try to shape NHS reform, attending campaign meetings and writing to national newspapers to express their views. Poor quality consultative encounters have made members of the public cynical, but it is not necessarily too late to re-engage them in a productive and new ways.

To do so, policymakers must recognize that “the public” are not an uncomplicated nor a homogenous mass, who will automatically, and without reflection, object to any type of NHS reform. Historically, public views around the NHS have long been complex and varied. This was the case at the inception of the NHS when—as we have seen—some members of the public were simply disinterested, whereas others were passionate advocates for the new service, interested in reading, interpreting, and contributing to relevant policy documents. Since 1945, members of the public have sought to engage with NHS policy in a variety of ways. Underlying overall strong support from the NHS, publics have had a range of experiences of different elements of primary, secondary, and community care functioning across various locales.

In this context, policymakers should also recognize that members of the public hold valuable expertise in terms of, for example, advising on how equal access to healthcare can be promoted, and in explaining why public health and prevention initiatives may not always be taken up effectively. Members of the public have developed this expertise through voluntary and paid work, and as patients, friends, members of families and communities, who have come in to contact with the service on multiple occasions over their lifetimes. Accessing these public views is worthwhile, but requires meaningful, careful, and accessible engagement with a range of public groups, and the payment of careful attention to the cultural and social contexts within which healthcare operates. Policymakers have been especially wary of engaging with campaigns around hospital closures. Yet such campaigns reflect public investment and belief in the principles and potential of the NHS. Anti-closure campaigns often also reflect valid concerns about whether particular sites are the only spaces in which certain communities can access healthcare. Such campaigns are significant, and historically relatively frequent: they must be addressed and engaged with, not ignored.

## Conclusion

Current debates among policymakers and think-tanks about public consultation are too present-focused, with public involvement in NHS decision-making constructed as “new,” or dating back only as far as the 1990s. By thinking about consultation in its longer historical contexts, two points become clear. First, thinking about consultation as “new” is potentially a means through which to devalue it; to construct it as an unnecessary, inappropriate, transient, or faddish part of NHS practice. However, looking to history belies this interpretation. Consultation has in fact been part of the theory and practice of postwar health policy since the inception of the NHS. A second issue which becomes clear, in looking to history, is that official consultative mechanisms have never been able to slake the public thirst for engagement. This does not necessarily mean that state-run consultative processes have always been ill-conceived. Rather, formal mechanisms will never provide the only source of public input. Indeed, there are—and have been from its inception—a broad array of publics interested in NHS reform, including people conceptualizing themselves as patients, family members, citizens, and members of communities, and acting individually or through voluntary organizations. Accordingly, public attempts to participate in shaping reform have included participation in consultative procedures, marches, rallies, petitions, and the formation of campaign groups, as well as electoral behavior.

Policymakers have likewise used a range of consultative mechanisms, encompassing hospital services, primary care settings, and community services, administered over time by an array of different management structures organized by local and national authorities. Although this article focused on England, the NHS itself has also comprised, throughout the late twentieth century, the four health systems managed separately for England, Wales, Scotland, and Northern Ireland, each accountable to different national politicians and departments. Seeking to justify, progress, or advise their reforms, the NHS and successive Governments have variously: informed the public about change; sought out feedback on predetermined decisions; worked with community members over time to ensure that their concerns are addressed before reform; and devolved decisions into the hands of individuals.^45^

Given the variety and depth of public opinion and feeling about the NHS, health authorities should take a broad approach to consultation. Even though policymakers have engaged through a range of institutions, and have also increasingly discussed the significance of consultation since the 1970s, many members of the public have felt—and continue to feel—deeply concerned about the future of the NHS. Consultative bodies have increasingly listened to public voices, but politicians have not always shaped policy accordingly. In part, this reflects the challenges for democratic government in terms of understanding and representing varied public interests. At the same time, politicians have historically also expressed deeply held suspicions of consultative processes in relation to the NHS. Policymakers have stated that these may create overly complex and confused systems, and that public affection for the NHS may be a barrier to reform, rather than a benefit. These attitudes, as well as fundamental power imbalances between publics and policymakers, have shaped and tainted consultative encounters. Memories of cases when consultative processes were ignored, absent, or ineffective have been passed down between families, communities, and campaign groups, and are magnified through media analysis.

Even though policymakers and publics have had differing opinions about the service, both groups have emphasized a cultural vision of the service as “our NHS,” belonging to, and meaningful to, everyone. The vision of the service as a national one, belonging to “the people,” reflects and shapes public interest in NHS reform, and has created the expectation that policymakers will and should consult as broadly as possible. Public interest in the NHS is long standing, and is not going away. Poor quality consultative procedures, or the absence of such work, will contribute to public distrust in politicians, as well as leading to legal challenges and public and professional outcry.

In this context, and as heated debate continues about NHS reforms and funding, policymakers should engage members of the public far more effectively, recognizing the wealth of expertise and experiences held by patients, community groups, and NHS workers who use, volunteer for, run, donate to, and indeed love the service every day. Engaging effectively with these groups is time-consuming, and requires long-standing and regular points of contact with multiple groups, enabling a diverse range of individuals to contribute to debate in appropriate and accessible ways. Nonetheless, such consultation may also provide a useful model for best practice in responding to public preferences and demands, and in terms of understanding how to deploy and utilize public affection and enthusiasm to the benefit of national systems.

Consultative practice will not evolve quickly or uniformly, but history also shows that individual politicians and health authorities can themselves make a difference by critically assessing and reshaping their consultative procedures on a local level, and by engaging the public as partners in promoting good health. These policymakers can also fund, support and listen to broad range of voluntary and mediatory groups, established to consult with, represent, and empower a diverse range of communities. Such varied voluntary organizations—patient groups, campaigners, community groups, staff groups—have always had, and continue to play, a key role in analyzing, bringing to light, challenging and contributing to the formation of NHS policy and practice today.

## Footnotes

^1^ For a detailed account of the 2012 Act see: Nicholas Timmins, *Never Again?: The Story of the Health and Social Care Act 2012* (London: The Institute for Government and the King’s Fund, 2012).

^2^ Harry Evans and Dan Wellings, “What Does the Public Think about the NHS?,” *The King’s Fund*, September 16, 2017, https://www.kingsfund.org.uk/publications/what-does-public-think-about-nhs.

^3^ George Gosling, *Payment and Philanthropy in British Healthcare, 1918–48* (Manchester: Manchester University Press, 2017).

^4^ Ben Jackson, “Why Was the Beveridge Report So Popular?,” University of Oxford podcasts, June 22, 2015, https://podcasts.ox.ac.uk/why-was-beveridge-report-so-popular.

^5^ Ministry of Health, *A National Health Service: The White Paper Proposals in Brief* (London: Her Majesty’s Stationary Office, 1944), 3.

^6^ Geoffrey Rivett, “Bevan and the NHS, 1945–1948,” NHS History Net, http://www.nhshistory.net/bevan.htm.

^7^ Rivett, “Bevan and the NHS” (Ref. 6).

^8^ Jack Saunders, “The British Medical Association,” People’s History of the NHS, http://peopleshistorynhs.org/encyclopaedia/the-british-medical-association/.

^9^ Socialist Medical Association, “Your New Health Service,” 1946, 2, Modern Records Centre, Coventry (hereafter MRC), 126/TG/377/1/1/34.

^10^ The opposition of the Medical Practitioners Union in 1944 was based on criticism that the Service: excluded industrial health and school children; failed to make the Ministry of Health dedicate its time, specifically, to health (in addition to local government work); and promoted the treatment of disease, rather than promotion of good health. (“Mr Willink’s Lost Opportunity,” Medical Practitioners Union, April 1944, https://www.sochealth.co.uk/national-health-service/the-sma-and-the-foundation-of-the-national-health-service-dr-leslie-hilliard-1980/mr-willinks-lost-opportunity-mpu-april-1944/.) For the 1948 circular by the MPU, see L. W. Hefferman, “National Health Service: You Must Decide,” circular, January 1948, 1, Trade Union Congress, Medical Practitioners Union, MRC, 292/847/5/126.

^11^ Ministry of Health, The National Health Service (London: Her Majesty’s Stationery Office, 1948), 14, MRC, 21/1489.

^12^ Hefferman, “National Health Service” (Ref. 10).

^13^ Charles Hill, “Your Doctor—and You,” *Daily Mail*, August 29, 1945, 2.

^14^ Charles Hill, “50,000 Doctors Say, ‘The Plan Won’t Work,’” *Daily Mail*, March 22, 1946, 2.

^15^ Nick Hayes, “Did We Really Want a National Health Service?: Hospitals, Patients and Public Opinions before 1948,” *English Historical Review* 127, no. 526 (2012), 625–61, on 639.

^16^ Roberta Bivins, “The Appointed Day: Celebrated or Silent?,” People’s History of the NHS, https://peopleshistorynhs.org/the-appointed-day-celebrated-or-silent/.

^17^ See: Philip Begley, Sally Sheard, and Eleanor Mackillop, eds. *The 1974 NHS Reorganisation: The Transcript of a Witness Seminar Held at the University of Liverpool in London on 9 November 2016* (Liverpool: Department of Public Health and Policy, University of Liverpool, 2017), https://www.liverpool.ac.uk/media/livacuk/instituteofpsychology/researchgroups/GoH,The,1974,NHS,Reorganisation.pdf, 6.

^18^ Begley, Sheard, and Mackillop, eds., *1974 NHS Reorganization* (Ref. 17), 15.

^19^ Alex Mold, *Making the Patient-Consumer: Patient Organisations and Health Consumerism in Britain* (Manchester: Manchester University Press, 2015), 50.

^20^ Christine Hogg, *Citizens, Consumers and the NHS: Capturing Voices* (Basingstoke: Palgrave Macmillan, 2008), 22–23.

^21^ Hogg, *Citizens* (Ref. 20), 23.

^22^ National Health Service Reorganisation Act, 1973, c. 32, 10.

^23^ Mold, *Making the Patient-Consumer* (Ref. 19), 53.

^24^ Melanie Phillips, “Hospital Closure ‘Hurried,’” *Guardian*, November 13, 1979, 4.

^25^ 975 Parl. Deb. H.C. (5th ser.) (1975) col. 1706.

^26^ Peter Hildrew, “Health Authority Wins Test Case on Hospital Closure,” *Guardian*, October 3, 1986, 3.

^27^ Mold, *Making the Patient-Consumer* (Ref. 19), 50.

^28^ Hogg, *Citizens* (Ref. 20), 23.

^29^ Christine Hogg (Secretary of a London CHC from 1974 to 1980, independent consultant for various CHCs, the Association of CHCs for England and Wales, and the Society of CHC staff from 1984), cited in Mold, *Making the Patient-Consumer* (Ref. 19), 55.

^30^ See the London Health Emergency archival collections at the MRC.

^31^ “Health Authorities Rapped for Closing Bone Marrow Unit,” *British Medical Journal* 306, no. 6894 (1993): 1712.

^32^ Sarah Hall, “Pensioner Wins Court Battle to Stop US Healthcare Giant,” *Guardian*, August 24, 2006, https://www.theguardian.com/society/2006/aug/24/health.politics.

^33^ Mold, *Making the Patient-Consumer* (Ref. 19).

^34^ Mold, *Making the Patient-Consumer* (Ref. 19).

^35^ Timmins, *Never Again?* (Ref. 1), 14–15.

^36^ Rudolf Klein and Bill New, *Two Cheers?: Reflections on the Health of NHS Democracy* (London: King’s Fund, 1998), 9.

^37^ Klein and New, *Two Cheers?* (Ref. 36), 9.

^38^ Point made verbally in discussions by Professor Roberta Bivins, University of Warwick.

^39^ “NHS: Fortieth Anniversary, the NHS Background,” August 1988, Conservative Medical Society Bulletin, Issue No. 11, 1, Bodleian Library, Conservative Party Archives.

^40^ House of Commons Liaison Committee, The Prime Minister, Oral and Written Evidence, Tuesday February 7, 2006, Tony Blair, 45.

^41^ Martin Gorsky and Virginia Preston, eds., *Tomlinson Report and After: Reshaping London’s Health Services 1992–1997*, Transcript of a Witness Seminar, November 13, 2012, 27–29, 41.

^42^ “Barking Strike: Spread the Action,” *Health Emergency*, June 1984, 7; “Rain On Our Parade,” *Health Emergency*, July–August 1984, 2; “Barking: The Fight Goes On!,” *Health Emergency*, August 1985, 7.

^43^ Politics of Health Group, Politics of Health 6: February 1983, Letters, 14, Wellcome Library, SA/PHG/2/1-7.

^44^ Janet, “Are You Listening to Us, Tony Blair?,” *Daily Mail*, April 18, 2002, 63.

^45^ See for reference how NHS England uses the “Ladder of Engagement and Participation,” based on the work of Sherry Arnstein, NHS England, “Patient and Public Participation Policy,” https://www.england.nhs.uk/publication/patient-and-public-participation-policy/, 14.

